# Repression of Middle Sporulation Genes in *Saccharomyces cerevisiae* by the Sum1-Rfm1-Hst1 Complex Is Maintained by Set1 and H3K4 Methylation

**DOI:** 10.1534/g3.117.300150

**Published:** 2017-10-24

**Authors:** Deepika Jaiswal, Meagan Jezek, Jeremiah Quijote, Joanna Lum, Grace Choi, Rushmie Kulkarni, DoHwan Park, Erin M. Green

**Affiliations:** * Department of Biological Sciences, University of Maryland Baltimore County, Maryland 21250; † Department of Mathematics and Statistics, University of Maryland Baltimore County, Maryland 21250

**Keywords:** histone methylation, Set1, gene expression, sporulation, meiosis

## Abstract

The conserved yeast histone methyltransferase Set1 targets H3 lysine 4 (H3K4) for mono, di, and trimethylation and is linked to active transcription due to the euchromatic distribution of these methyl marks and the recruitment of Set1 during transcription. However, loss of Set1 results in increased expression of multiple classes of genes, including genes adjacent to telomeres and middle sporulation genes, which are repressed under normal growth conditions because they function in meiotic progression and spore formation. The mechanisms underlying Set1-mediated gene repression are varied, and still unclear in some cases, although repression has been linked to both direct and indirect action of Set1, associated with noncoding transcription, and is often dependent on the H3K4me2 mark. We show that Set1, and particularly the H3K4me2 mark, are implicated in repression of a subset of middle sporulation genes during vegetative growth. In the absence of Set1, there is loss of the DNA-binding transcriptional regulator Sum1 and the associated histone deacetylase Hst1 from chromatin in a locus-specific manner. This is linked to increased H4K5ac at these loci and aberrant middle gene expression. These data indicate that, in addition to DNA sequence, histone modification status also contributes to proper localization of Sum1. Our results also show that the role for Set1 in middle gene expression control diverges as cells receive signals to undergo meiosis. Overall, this work dissects an unexplored role for Set1 in gene-specific repression, and provides important insights into a new mechanism associated with the control of gene expression linked to meiotic differentiation.

The control of gene expression in response to environmental signals is regulated by the concerted activity of transcription factors, histone-modifying enzymes, and chromatin remodelers, among other proteins and regulatory RNAs (Suganuma and Workman 2011; Allis and Jenuwein 2016; Jaiswal *et al.* 2017). In diploid *Saccharomyces cerevisiae*, the transition from mitotic growth to meiotic divisions and spore formation is initiated when cells encounter limiting levels of glucose and nitrogen in the presence of a nonfermentable carbon source (Neiman 2011; Jaiswal *et al.* 2017). The morphological and physiological changes underlying meiosis and spore formation are driven by a highly regulated gene expression cascade, for which the genes have been broadly grouped into temporal classes including early-, middle- and late-expressing genes (Chu *et al.* 1998; Primig *et al.* 2000). As these genes are specialized for the processes of meiosis and sporulation, their expression is largely repressed during vegetative growth. In multiple systems, including flies and human cells, repression of meiosis-specific genes prevents aberrant chromosome segregation and maintains genome stability of somatic cells (Janic *et al.* 2010; Folco *et al.* 2017). It is therefore critical to understand the chromatin landscape that promotes repression of meiotic differentiation genes.

In particular, repression of middle sporulation genes during yeast vegetative growth has been well-characterized to be mediated by the Sum1-Rfm1-Hst1 complex (Xie *et al.* 1999; McCord *et al.* 2003). Sum1 is a DNA binding protein that recognizes the middle sporulation element (*MSE*) in the promoters of middle sporulation genes (Ozsarac *et al.* 1997; Xie *et al.* 1999), and represses expression through the recruitment of the histone deacetylase (HDAC) Hst1, which is linked to Sum1 via the adaptor protein Rfm1 (Xie *et al.* 1999; McCord *et al.* 2003; Robert *et al.* 2004). Sum1 is also thought to repress a subset of genes through an Rfm1/Hst1-independent pathway, although the mechanism for this is not clear (McCord *et al.* 2003; Corbi *et al.* 2014). One means by which repression of middle genes is relieved during meiotic progression is through the phosphorylation of Sum1 by the kinases Ime2 and Cdk1 (Pak and Segall 2002; Ahmed *et al.* 2009; Shin *et al.* 2010). This promotes removal of Sum1 from the promoter of *NDT80*, a master regulator that encodes a transcription factor, which ultimately displaces Sum1 from downstream middle gene promoters (Pierce *et al.* 2003; Shin *et al.* 2010; Corbi *et al.* 2014). While progress has been made in characterizing the activation of middle sporulation genes in meiosis, the extent to which chromatin-based mechanisms contribute to repression of these genes during vegetative growth is still unclear.

The yeast enzyme Set1, the catalytic component of the COMPASS complex, performs mono, di, and trimethylation of lysine 4 (K4) of histone H3 (Miller *et al.* 2001; Roguev *et al.* 2001; Nagy *et al.* 2002). Set1 and H3K4 methylation are commonly linked to active gene expression due to the cotranscriptional recruitment of Set1 to RNA pol II, and the association of H3K4 methyl species with actively transcribed genes (Bernstein *et al.* 2002; Santos-Rosa *et al.* 2002; Ng *et al.* 2003; Liu *et al.* 2005; Pokholok *et al.* 2005; Kirmizis *et al.* 2007). However, loss of Set1 in budding yeast leads to a higher proportion of genes whose expression is upregulated rather than downregulated (Venkatasubrahmanyam *et al.* 2007; Guillemette *et al.* 2011; Lenstra *et al.* 2011; Margaritis *et al.* 2012; Weiner *et al.* 2012; Martín *et al.* 2014). The majority of upregulated genes are subtelomeric, and normally silent or lowly expressed. Additionally, genes that are repressed under normal, log-phase growth conditions in wild-type cells, including galactose-induced genes, phosphate-responsive genes, ergosterol biosynthetic genes, and sporulation genes, show increased expression in *set1Δ* cells (Carvin and Kladde 2004; Wang *et al.* 2011; Margaritis *et al.* 2012; South *et al.* 2013; Ramakrishnan *et al.* 2016). Set1 also acts to repress ribosomal protein genes under stress conditions (Weiner *et al.* 2012). Similarly, in *Schizosaccharomyces pombe*, cells lacking Set1 exhibit derepression of heterochromatin-associated loci, such as *Tf2* retrotransposons and pericentromeric repeats, as well as stress-response genes (Lorenz *et al.* 2014; Mikheyeva *et al.* 2014).

For some of these gene classes, different mechanisms have been proposed to describe how Set1 promotes repression. In the case of subtelomeric genes, loss of silencing in *set1Δ* cells has been proposed to be due to titration of the Sir protein complex, which deacetylates H4K16, away from subtelomeric chromatin (Santos-Rosa *et al.* 2004; Venkatasubrahmanyam *et al.* 2007). However, data regarding other functional roles for Set1, including its links to noncoding RNA production and genome stability pathways (Corda *et al.* 1999; Margaritis *et al.* 2012; Jezek *et al.* 2017), suggest alternate mechanisms for Set1-mediated repression. Interestingly, Set1-dependent repression in fission yeast appears to occur through both H3K4 methylation-dependent and independent mechanisms, and it works together with the histone deacetylase Clr3 to promote repression (Lorenz *et al.* 2014). Set1 has also consistently been linked to gene repression through noncoding antisense transcription, often originating at the 3′ end of coding regions, such as for *GAL* and *PHO* genes (Berretta *et al.* 2008; Camblong *et al.* 2009; van Dijk *et al.* 2011; Kim *et al.* 2012; Margaritis *et al.* 2012; Castelnuovo *et al.* 2013, 2014). Although multiple mechanisms of repression have been proposed in these cases, one example is the cotranscriptional deposition of H3K4 methyl species during noncoding transcription, which recruits repressive chromatin complexes through methyl-lysine effector proteins, such as the HDAC-containing Set3C (Kim and Buratowski 2009; Kim *et al.* 2012). Together, these studies have highlighted potentially diverse means by which Set1 promotes gene repression, and indicate that it can act as a principal regulator of specific functional classes of genes under certain environmental conditions.

Here, we investigated the role for Set1 and H3K4 methylation in the repression of middle sporulation genes during vegetative growth. Interestingly, Set1 appeared to promote the association of the sequence-specific DNA binding protein Sum1 and the HDAC Hst1 with middle sporulation genes to maintain deacetylation, primarily at H4K5, and gene repression. Our results suggest a model in which Set1 establishes a chromatin state that specifically maintains Sum1 and Hst1 at a subset of middle sporulation loci, and in its absence, gene-specific derepression occurs. In conjunction with characterizing this repressive role for Set1, our findings further indicate that the Sum1 repressive complex partially depends on histone methylation status, in addition to DNA sequence elements, to maintain optimal repression.

## Materials and Methods

### Yeast strains, plasmids, and growth conditions

The genotypes for all *S. cerevisiae* strains used in this study are listed in Table 1. Standard growth media were used as appropriate, including YPD (1% yeast extract, 2% peptone, 2% dextrose) and synthetic complete (SC) or dropout media (US Biological). Strains carrying gene deletions or epitope-tagged alleles were constructed using insertion of targeted PCR cassettes amplified from the pFA6a vector series (Longtine *et al.* 1998). Double mutant strains were isolated by haploid mating, sporulation, and tetrad dissection. The N-terminal 3xFLAG-tagged *SET1* was generated using PCR amplification from plasmid pZM467 and subsequent loop-out of the *URA3* marker by selection on plates containing 5-fluoroorotic acid (Moqtaderi and Struhl 2008). Strains were confirmed by growth on selective media and colony PCR using primers specific to individual gene deletions or epitope tag insertions. Expression of epitope-tagged proteins was validated by Western blotting. The *pRS316-SET1* expression vector was generated by cloning a PCR fragment that amplified the *SET1* locus, including its promoter and 3′UTR, into *pRS316*.

**Table 1:** Yeast strains used in this study

Strain Number	Genotype	Reference
yEG001	*MATa his3Δ1 leu2Δ0 met15Δ0 ura3Δ0* (isogenic to BY4741)	YKO
yEG230	*MATα his3Δ1 leu2Δ0 met15Δ0 ura3Δ0* (isogenic to BY4742)	Jezek *et al.* (2017)
yEG232	*MATa set1Δ*::*KANMX*	Jezek *et al.* (2017)
yEG100	*MATa spp1Δ*::*KANMX*	Jezek *et al.* 2017)
yEG110	*MATa sdc1Δ*::*KANMX*	Jezek *et al.* 2017)
ySL151	*MATa his3*Δ*200 leu2*Δ*1 ura3-52 trp1*Δ*63 lys2-128δ hht1-hhf1*Δ:*LEU2 hht2-hhf2*Δ:*HIS3* p*TRP1-HHT2-HHF2*	Krishnamoorthy *et al.* (2006)
ySL171	*MATa his3*Δ*200 leu2*Δ*1 ura3-52 trp1*Δ*63 lys2-128δ hht1-hhf1*Δ:*LEU2 hht2-hhf2*Δ:*HIS3* p*TRP1-hht2K4A-HHF2*	Krishnamoorthy *et al.* (2006)
yEG349	*MATa set3Δ*::*NATMX*	This study
yEG384	*MATa jhd1Δ*::*KANMX*	This study
yEG623	*MATα rad6Δ*::*HIS3MX*	This study
yEG596	*MATα hst1*::*HST1-MYC*::*HIS3MX*	This study
yEG603	*MATα hst1*::*HST1-MYC*::*HIS3MX set1Δ*::*KANMX*	This study
yEG613	*MATα sum1*::*SUM1-MYC*::*HIS3MX*	This study
yEG619	*MATα sum1*::*SUM1-MYC*::*HIS3MX set1Δ*::*KANMX*	This study
yEG630	*MATα sum1Δ*::*HIS3MX*	This study
yEG631	*MATα sum1Δ*::*HIS3MX set1Δ*::*KANMX*	This study
yEG032	*MATa/α leu2*::*hisG/leu2*::*hisG trp1*::*hisG/trp1*::*hisG lys2-SK1/lys2- his4-N/his4-G ura3-SK1/ura3-SK1 ho*::*LYS2/ho*::*LYS2* (SK1)	Krishnamoorthy *et al.* (2006)
yEG625	*MATa/α set1Δ*::*KANMX/set1Δ*::*NATMX* (SK1)	This study
yEG645	*MATα* yEG230 *+* *pRS316*	This study
yEG646	*MATα* yEG230 *+* *pRS316-SET1*	This study
yEG647	*MATa set1Δ*::*KANMX + pRS316*	This study
yEG648	*MATa set1Δ*::*KANMX + pRS316-SET1*	This study
yEG643	*MATa set1*::*FLAG-SET1*	This study

All strains are derived from the BY4741 or BY4742 (shown for yEG001 and yEG230) genetic backgrounds, except yEG032 and yEG625, which are from the SK1 genetic background. Strains obtained from the Yeast Knockout Collection are indicated with YKO.

### Genome-wide expression data analysis

Statistical analyses were performed using the R statistical computing environment. Datasets include previously published and analyzed RNA-sequencing experiments of *set1Δ* cells (Martín *et al.* 2014; Jezek *et al.* 2017; GEO accession number GSE52086) and publically available microarray datasets from Kemmeren *et al.* 2014 (GEO accession number GSE42527, http://deleteome.holstegelab.nl/). Gene ontology (GO) analysis was performed using FunSpec (Robinson *et al.* 2002). Temporal classes of gene expression during sporulation were previously identified (Chu *et al.* 1998), and gene expression of all genes within each class was determined from genome-wide datasets as described in the *Results*. Differences between classes for tested mutants were evaluated using unequal variance *t*-tests (Welch’s *t*-tests; Welch 1947) with a Bonferroni correction.

### Quantitative reverse transcriptase PCR

The Masterpure Yeast RNA Purification kit (Epicentre) was used to extract total RNA from 1.5 ml of an OD_600_ ∼0.6–0.8 culture of yeast cells. The Turbo DNA-free kit (Ambion) was used to eliminate genomic DNA, and cDNA was generated from 1 µg of total RNA using the iScript cDNA Synthesis kit (Bio-Rad) containing both oligo dT and random hexamers for priming reverse transcription. For quantitative PCR (qPCR) of transcript levels, 0.5 µl of the cDNA mixture was added to 1X iTAQ Universal SYBR Green Supermix (Bio-Rad) with gene-specific primers in a 10 µl reaction. (Primer sequences listed in Supplemental Material, Table S1 in File S1.) A Bio-Rad CFX384 Real-time Detection System was used for amplification. Three technical replicates were performed for each reaction, and a minimum of three biological replicates was performed for each experiment. Gene expression values were determined relative to the control gene *TFC1*, whose expression is reported to be stable under different growth conditions (Teste *et al.* 2009).

### Immunoblotting

Yeast lysates were prepared by 0.2 M NaOH treatment of harvested cells (Kushnirov 2000), followed by SDS-PAGE and transferred to PVDF. The following primary antibodies were used: rabbit anti-H3K4me3 (catalog no. 39159; Active Motif), rabbit anti-H3K4me2 (catalog no. 39141; Active Motif), rabbit anti-H3 (catalog no. 39163; Active Motif), mouse anti-MYC (clone 9E10, catalog no. MA1980; Invitrogen). A Licor C-DiGit Chemiluminescent Western Blot Scanner was used for imaging.

### Sporulation assays and gene expression timecourse

Diploid SK1 cells were used for sporulation assays and gene expression analysis, as described (Sollier *et al.* 2004). Briefly, log phase cells from YPD were transferred to YPA (1% yeast extract, 2% peptone, 1% potassium acetate) and grown at 30° with shaking for 8 hr, then diluted to 2 × 10^6^ cells/ml and grown in YPA for an additional 14 hr. Cells were harvested, washed with sterile water, and transferred to sporulation medium (SPM; 1% potassium acetate supplemented with 25% amino acids) and incubated at 30° with shaking. The sporulation rate was determined at 24 hr using light microscopy to count asci. For gene expression analysis, 1.5 ml of cells were collected from YPD, YPA just before transfer to SPM (0 hr timepoint), and at 4 and 8 hr after transfer into SPM. RNA extraction and qRT-PCR were performed as described above.

### Chromatin immunoprecipitation

Chromatin immunoprecipitation (chIP) was performed as described (Meluh and Broach 1999; Liu *et al.* 2005; Jezek *et al.* 2017). Briefly, 100 ml of mid-log phase yeast were fixed with 1% formaldehyde for 30 min. Extracts were generated by bead beating and chromatin was digested with micrococcal nuclease. Chromatin extracts were normalized to total protein content. Antibody was prebound to protein A/G magnetic beads (Pierce) overnight, and then added to the extracts and rotated 3 hr or overnight at 4°. Protein-DNA complexes were eluted using 1% SDS and 0.1 M NaHCO_3_, cross-links were reversed, and samples were treated with proteinase K and RNaseA. DNA was extracted with phenol-chloroform-isoamyl alcohol, and precipitated using ethanol. qPCR was performed as described above using 0.5 μl chIP DNA per reaction and primers specific to unique genomic regions. (Primer sequences listed in Table S1 in File S1.) Technical triplicates were performed for each qPCR reaction and three biological replicates were performed for each chIP experiment. Percent input was calculated relative to 5% input. The following antibodies were used for chIP experiments: mouse anti-MYC (clone 9E10, catalog no. MA1980; Invitrogen), rabbit anti-H4K5ac (catalog no. ab51997; Abcam), rabbit anti-H4K12ac (catalog no. ABE532; EMD Millipore), rabbit anti-H4 (catalog no. 04-858; EMD Millipore), mouse anti-FLAG (catalog no. F1804; Sigma), rabbit anti-H3K4me2 (catalog no. 39142; Active Motif) and rabbit anti-H3 (catalog no. ab1791; Abcam).

### Data availability

Datasets used for genome-wide expression analyses are previously published (Kemmeren *et al.* 2014; Martín *et al.* 2014; Jezek *et al.* 2017) and publically available (GEO accession numbers GSE52086 and GSE42527). All other data necessary to support the conclusions of this work are represented fully within the article or in the supplemental material. All yeast strains and plasmids are available upon request.

## Results

### Set1 and H3K4 methylation repress middle sporulation genes during vegetative growth

**Figure 1:**
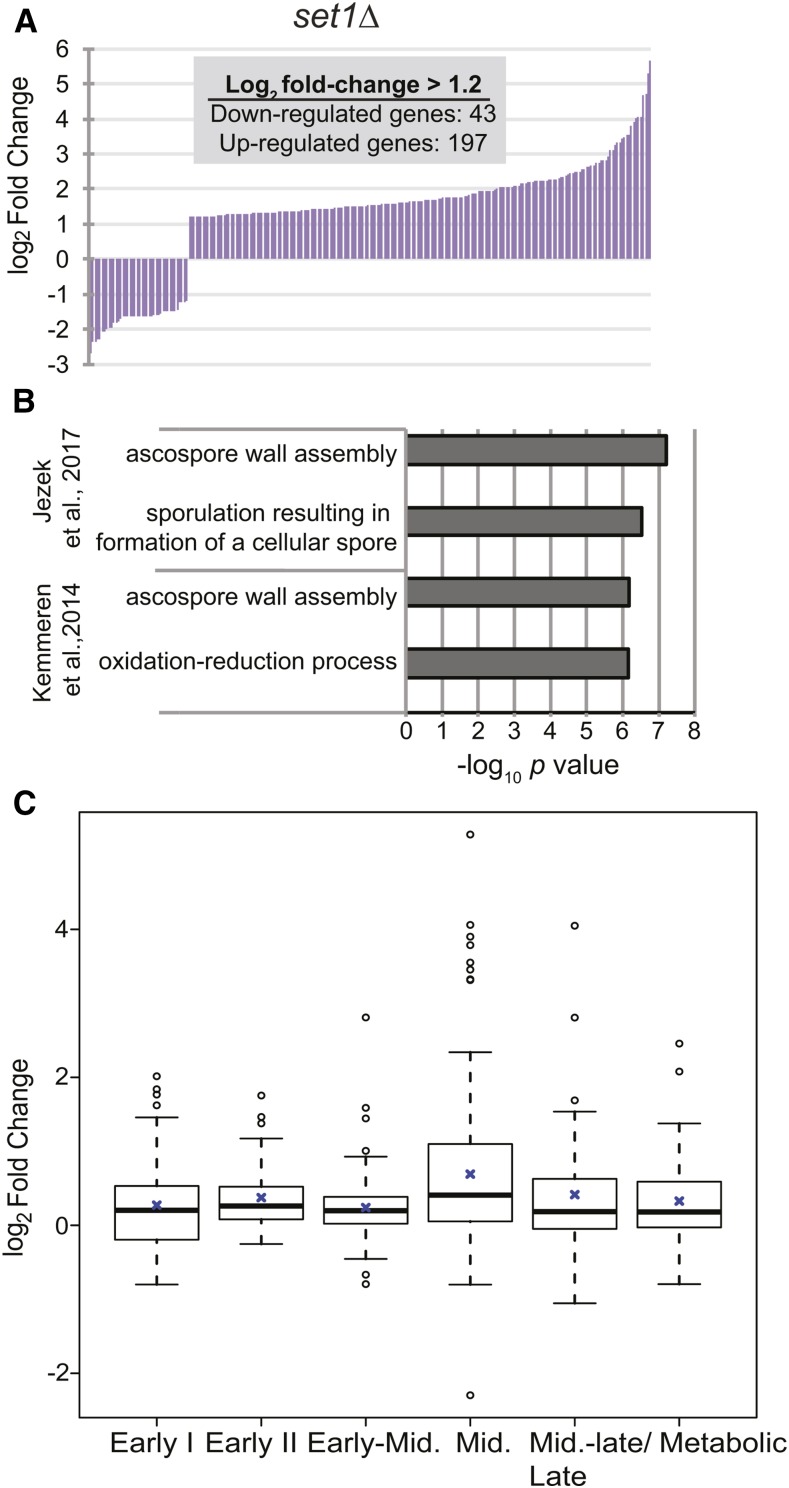
Set1 represses sporulation genes during vegetative growth. (A) Significantly differentially expressed (SDE) genes in *set1Δ* cells relative to wildtype from previously described RNA-sequencing experiments (Martín *et al.* 2014; Jezek *et al.* 2017). Graph represents all genes with log_2_ fold-change ≥1.2 and *p* ≤ 0.05. (B) −log_10_ *p*-value of GO terms enriched for the genes upregulated in *set1Δ* cells from Jezek *et al.* (2017), and from microarray data from Kemmeren *et al.* (2014). For GO analysis of the *set1Δ* microarray dataset, differentially expressed genes with log_2_ fold-change ≥0.5 (*p* ≤ 0.05) were used to analyze a similar number of genes (upregulated SDE genes = 197; downregulated SDE genes = 34) to those in the RNA-seq dataset. The discrepancy in log_2_ fold-change values is most likely attributed to the differing sensitivity for each method. (C) Log_2_ fold-change of genes from *set1Δ* cells belonging to each of the indicated sporulation classes, as previously defined (Chu *et al.* 1998). Due to a low number of genes categorized as late, these genes were combined with the middle-late class. Black line within boxes indicates median value and blue cross indicates mean value. Whiskers represent 1.5 times the interquartile range, with outliers plotted as circles.

Our previous RNA-sequencing (seq) analysis of haploid *set1Δ* mutants showed that the majority of differentially expressed genes in *set1Δ* are up-regulated relative to wildtype (Figure 1A; Martín *et al.* 2014; Jezek *et al.* 2017), consistent with other genome-wide studies of cells lacking Set1 grown under vegetative conditions (Venkatasubrahmanyam *et al.* 2007; Guillemette *et al.* 2011; Lenstra *et al.* 2011; Margaritis *et al.* 2012; Weiner *et al.* 2012; Kemmeren *et al.* 2014; Jezek *et al.* 2017). While a large fraction of the upregulated genes were telomere-associated (Venkatasubrahmanyam *et al.* 2007; Lenstra *et al.* 2011; Margaritis *et al.* 2012; Martín *et al.* 2014), GO analysis also identified genes linked to sporulation as the only enriched biological process within the dataset (Figure 1B). This is consistent with previously published reports of similar datasets (Guillemette *et al.* 2011; Lenstra *et al.* 2011; Margaritis *et al.* 2012), in which genes associated with sporulation were identified as upregulated in *set1Δ* cells. Likewise, GO analysis of another published dataset (Kemmeren *et al.* 2014) also showed enrichment for sporulation-associated genes among those genes upregulated in cells without Set1 (Figure 1B). The sporulation gene expression program in budding yeast has been categorized into at least seven temporal classes (Chu *et al.* 1998; Primig *et al.* 2000). We therefore tested whether or not genes upregulated in *set1Δ* cells were associated with specific temporal classes within the sporulation program. We observed statistically significant increased expression in the middle class of genes relative to all other classes (unequal variance *t*-test, *p*-value = 0.00013), whereas the other classes did not exhibit much change in expression in *set1Δ* cells (Figure 1C). Overall, genome-wide expression data has revealed a specific role for Set1 in repressing middle sporulation genes during vegetative growth.

**Figure 2:**
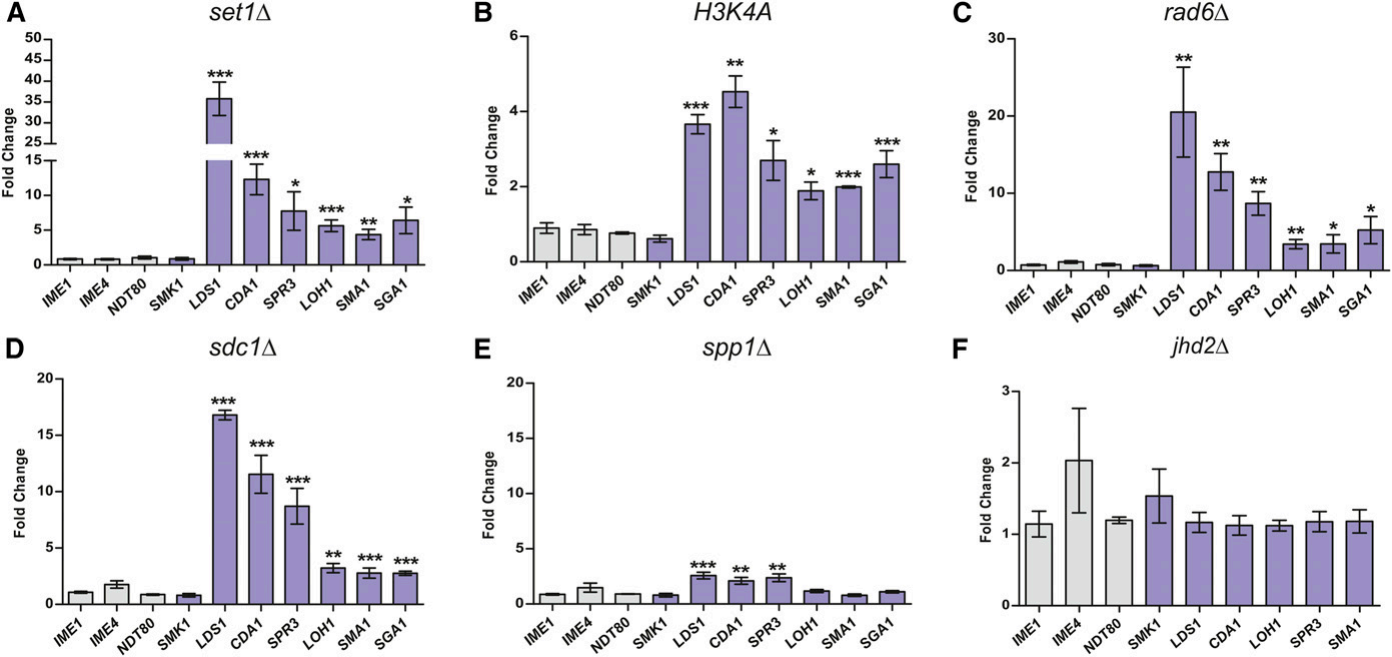
H3K4 methylation and COMPASS components are required for middle sporulation gene repression. Quantitative RT-PCR (qRT-PCR) of mRNA levels of early (gray) and middle (purple) sporulation genes. Expression values were determined relative to the housekeeping gene, *TFC1*, and the fold-change in each mutant is plotted relative to an isogenic wildtype strain for (A) *set1Δ*, (B) *H3K4A*, (C) *rad6Δ*, (D) *sdc1Δ*, (E) *spp1Δ*, and (F) *jhd2Δ*. Error bars represent SEM for a minimum of three biological replicates. Asterisks represent *p*-values from unpaired *t*-tests (* ≤ 0.05, ** ≤ 0.01, *** ≤ 0.001; no asterisk is not significant).

To further validate these results, and test the role of H3K4 methylation in derepression, we next used qRT-PCR to monitor the expression levels of a set of early and middle sporulation genes during vegetative growth in mutants that disrupt Set1 function or inhibit H3K4 methylation. Consistent with our findings from genome-wide analyses, we observed increased expression of the middle genes *LDS1*, *SMA1*, *CDA1*, *LOH1*, and *SPR3* in *set1Δ* cells (Figure 2A). As expected, there was no change in expression of the early genes *IME1* or *IME4*, or *NDT80*, characterized as an early-middle gene. Notably, the middle gene *SMK1* did not show any detectable derepression in *set1Δ* cells. The increased expression of middle genes in the *set1Δ* mutant was rescued in the presence of *SET1* expressed from a plasmid (Figure S1 in File S1), confirming that this phenotype is specifically due to the loss of Set1. We next assayed the same set of genes in cells carrying an *H3K4A* mutation, which blocks methylation by Set1. The cells expressing *H3K4A* also showed derepression of middle genes relative to cells with wildtype H3 (Figure 2B). Furthermore, disruption of H2B K123 monoubiquitination, which promotes H3K4 methylation, in *rad6Δ* mutants, resulted in increased expression of middle genes (Figure 2C). These data suggest that repression of middle genes relies on the methylation activity of Set1 on H3K4.

To further assess whether middle gene repression depends on mono, di, or trimethylation at H3K4, we used mutations in components of the COMPASS complex. *sdc1Δ* mutants are depleted for both H3K4me2 and H3K4me3, whereas *spp1Δ* is associated with loss of H3K4me3 only (Morillon *et al.* 2005; Schneider *et al.* 2005; Dehé *et al.* 2006). qRT-PCR of *sdc1Δ* and *spp1Δ* cells during vegetative growth showed significant derepression of middle genes in the absence of Sdc1; however, gene expression was mostly unchanged upon loss of Spp1 (Figure 2, D and E). This suggests that vegetative repression of middle sporulation genes is primarily dependent on H3K4me2 rather than H3K4me3. In addition, we found that loss of the H3K4 demethylase Jhd2 (Liang *et al.* 2007; Seward *et al.* 2007; Tu *et al.* 2007), which has been reported to have an overlapping role in expression regulation of certain genes with Set1 (Ramakrishnan *et al.* 2016), does not alter expression levels of middle sporulation genes under vegetative conditions (Figure 2F).

### The functional role of Set1 in gene expression control diverges with the onset of the sporulation program

**Figure 3:**
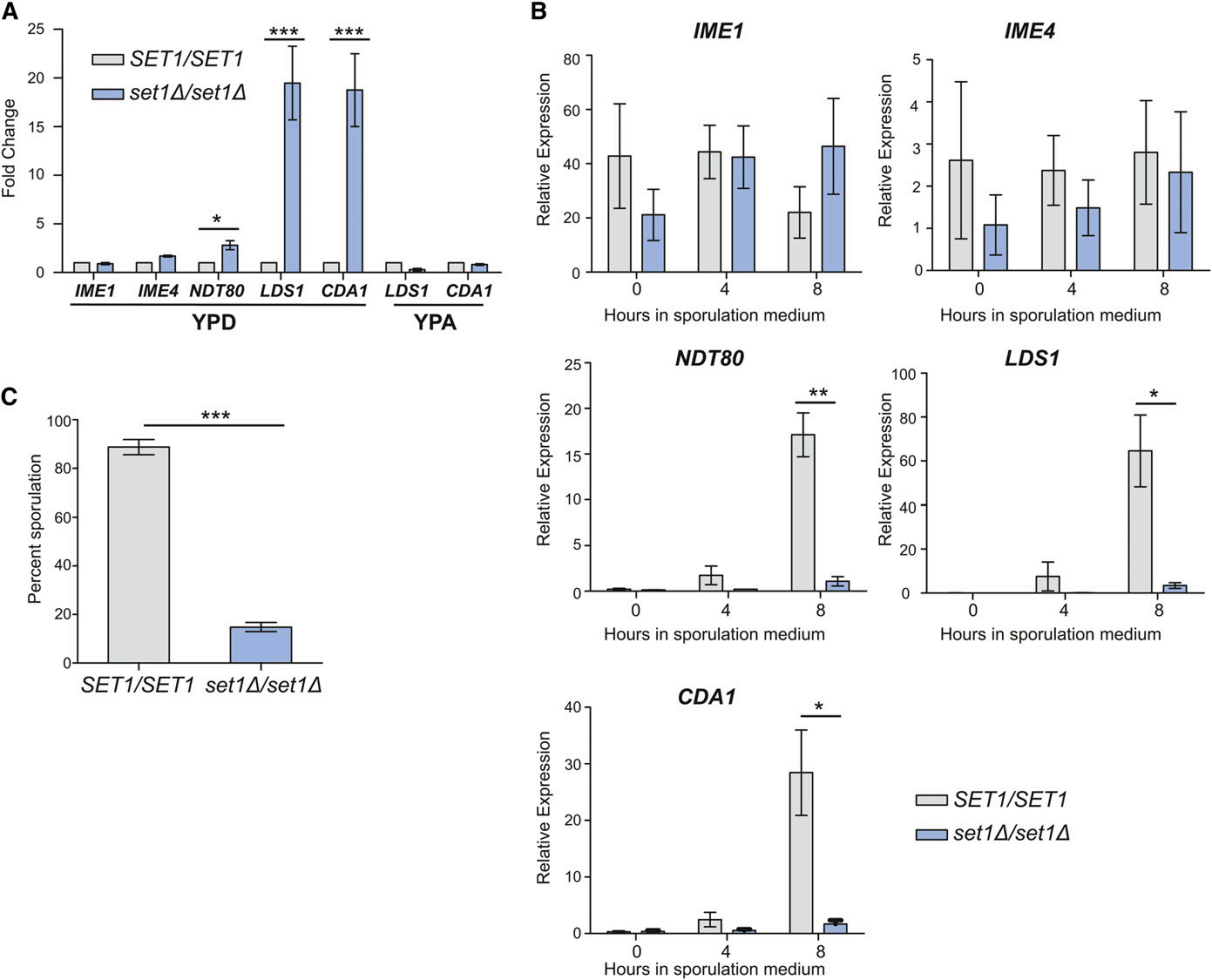
Set1 does not repress middle gene expression during sporulation. (A) Expression of early and middle genes by qRT-PCR in wildtype and *set1Δ MAT*a/α diploid yeast from the SK1 strain background grown in rich medium with glucose (YPD). Expression of the middle genes *LDS1* and *CDA1* are also shown following growth in rich medium with acetate (YPA). Expression levels were normalized to the control gene *TFC1* and fold-change was determined relative to wild type. Error bars represent SEM for three biological replicates. (B) Expression of early (*IME1* and *IME2*), early–mid (*NDT80*) and middle genes (*LDS1* and *CDA1*) of SK1 wildtype and *set1Δ* diploids at 0 (in YPA), 4, and 8 hr following transfer to SPM. Expression levels were normalized to the control gene *TFC1*. Error bars represent SEM for three biological replicates. (C) Percent sporulation of SK1 diploid cells following 24 hr in SPM. For all panels, asterisks represent *p*-values from unpaired *t*-tests (* ≤ 0.05, ** ≤ 0.01, *** ≤ 0.001; no asterisk is not significant).

Set1 has previously been linked to defective meiotic progression and spore formation (Nislow *et al.* 1997; Sollier *et al.* 2004), and the activation of middle sporulation genes during meiosis is delayed in the absence of Set1 and H3K4 methylation (Sollier *et al.* 2004). Here, we have analyzed Set1 and H3K4 methylation-mediated repression of middle sporulation genes in haploid cells dividing mitotically. Given that initiation of meiotic differentiation is regulated by a ploidy-sensing mechanism (Neiman 2011; Jaiswal *et al.* 2017), we investigated whether middle sporulation gene expression was different in *MAT*a/α diploid cells. In this case, we used cells from the SK1 strain background, which undergo rapid and synchronous sporulation upon nutrient deprivation. Consistent with our findings using BY4741 haploid yeast, we observed derepression of the middle genes *LDS1* and *CDA1* in *set1Δ/set1Δ* SK1 diploid cells, whereas there was only minor induction of *NDT80* and no change in expression of the early genes *IME1* and *IME4* under vegetative conditions (Figure 3A). We also followed the expression of these genes once cells were induced to undergo sporulation by transferring them to an acetate-containing medium, and, subsequently, SPM with acetate and limited nutrients. Interestingly, once cells were switched to rich medium containing acetate as the carbon source (YPA), middle sporulation genes were not derepressed in *set1Δ/set1Δ* diploids (Figure 3A). Furthermore, following transfer to nutrient-depleted sporulation medium, middle genes were not induced as the sporulation timecourse progressed (Figure 3B). In *set1Δ/set1Δ* diploids undergoing sporulation, the master regulator *NDT80*, which is required for the downstream activation of the middle genes *LDS1* and *CDA1*, was not expressed. We also monitored spore formation by visual inspection of asci at 24 hr after transferring cells to SPM. As expected, and previously reported (Nislow *et al.* 1997; Sollier *et al.* 2004), the *set1Δ/set1Δ* diploid cells were severely defective in spore formation (Figure 3C). Overall, these data suggest that while Set1 promotes repression of middle sporulation genes when cells are grown in the presence of a fermentable carbon source, this function is not required in a nonfermentable carbon source. It appears that as cells receive signals for inducing meiosis, the regulatory role of Set1 at middle genes is no longer necessary. Instead, meiotic functions for Set1, such as promoting proper recombination, are likely responsible for defects in *NDT80* induction and downstream middle gene activation, as well as the sporulation defects observed in *set1Δ/set1Δ* diploid cells (Sollier *et al.* 2004; Borde *et al.* 2009).

### Middle sporulation gene derepression in set1Δ does not depend on the Set3 histone deacetylase complex

The H3K4me2 mark has been linked to Set1-dependent repression by stabilizing the Set3 complex (Set3C), and its associated histone deacetylase (HDAC) activity at chromatin (Kim and Buratowski 2009; Kim *et al.* 2012). The Set3C contains the HDACs Hos2 and Hst1, of which Hst1 is also a component of the Sum1 repressive complex that targets middle sporulation genes (Pijnappel *et al.* 2001; McCord *et al.* 2003). We therefore investigated whether the derepression of middle genes in *set1Δ*, which appears dependent on H3K4me2 (Figure 2, D and E), is also dependent on intact Set3C. qRT-PCR analysis of *set3Δ* cells showed little change in both early and middle gene expression under vegetative conditions (Figure S2A in File S1). Additionally, we analyzed previously published microarray data of *set3Δ* cells grown under vegetative conditions (Kemmeren *et al.* 2014), and no significant changes in gene expression were observed among the different time classes of sporulation program genes (Figure S2B in File S1). Furthermore, a linear regression model showed no dependence of the *set1Δ* gene expression changes on *SET3* among either the middle genes, or any of the other sporulation time classes (Figure S2C in File S1). Our data therefore suggest that Set3 does not play a role in repression of middle genes under vegetative conditions, indicating that derepression in *set1Δ* mutants is not due to altered Set3C activity. While Set3C has previously been implicated in the regulation of early and middle sporulation genes, it was identified as a meiotic-specific repressor of these genes, and did not play a role during mitotic growth (Pijnappel *et al.* 2001), consistent with our findings.

### Set1 promotes binding of Sum1 and Hst1 and histone deacetylation at middle sporulation genes

Previously, we performed Spearman’s rank correlation between the gene expression profiles of *set1Δ* cells and mutants in over 700 transcription and chromatin-regulatory factors (Kemmeren *et al.* 2014; Jezek *et al.* 2017). In this analysis, we observed highly ranked correlations between *set1Δ* cells and mutants lacking components of the middle sporulation gene repressor complex containing Sum1, Rfm1, and Hst1 (Jezek *et al.* 2017). Lenstra *et al.* (2011) had also previously identified a similar correlation for *set1Δ* mutants. Given this correlation, and the known role for the Sum1-Rfm1-Hst1 complex in repressing middle sporulation genes during vegetative growth, we next investigated possible links between Set1 and this complex. We tested whether or not Set1 and the Sum1-Rfm1-Hst1 complex are likely to act in the same pathway using expression analysis of middle genes in *set1Δ* and *sum1Δ* single and double mutants. The *set1Δ sum1Δ* mutants showed similar derepression levels to the *sum1Δ* mutant (Figure S3 in File S1), suggesting an epistatic relationship, and indicating the possibility that Set1 and Sum1 are repressing middle genes through the same mechanism.

**Figure 4:**
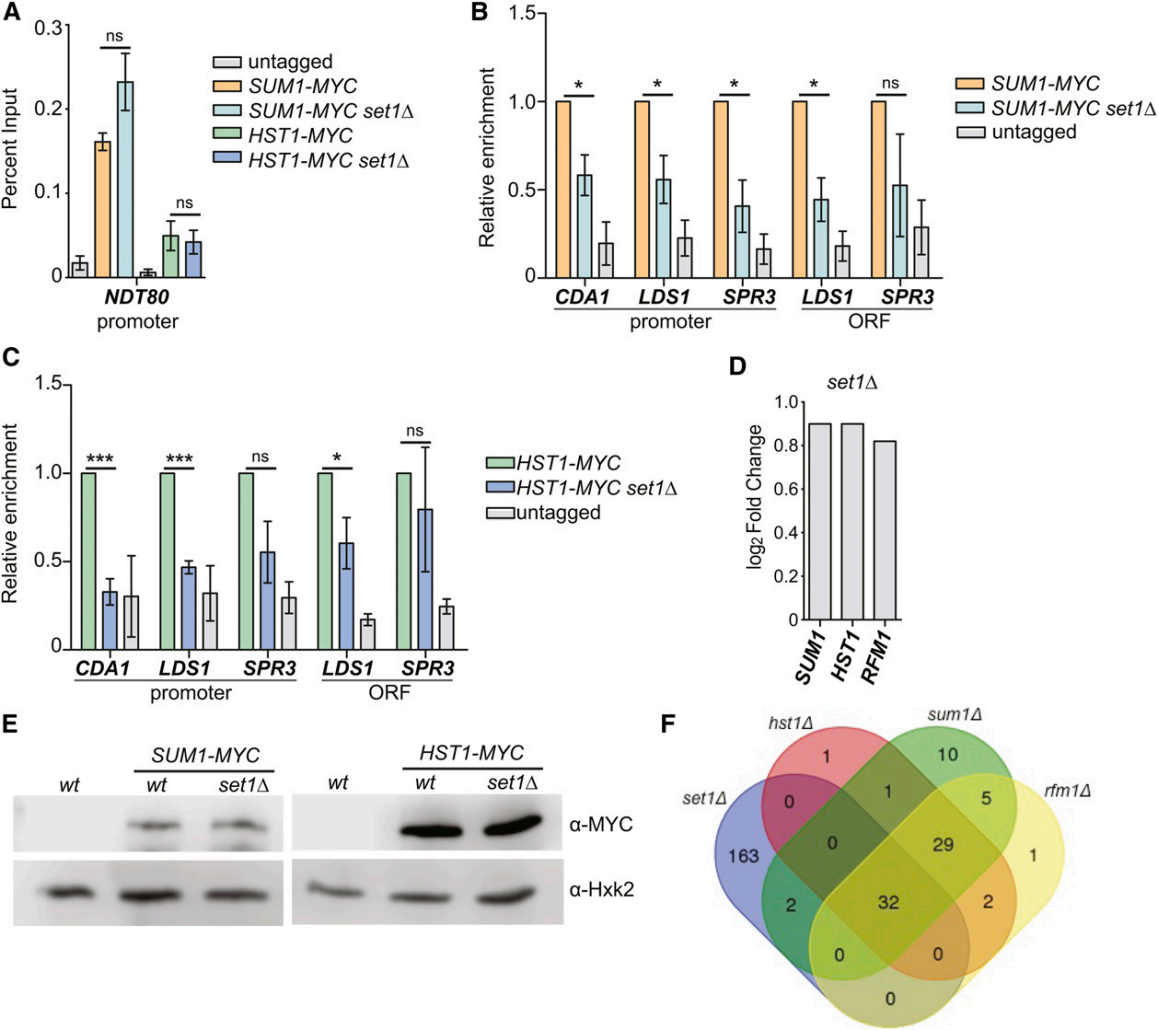
Sum1 and Hst1 are depleted from middle sporulation genes in *set1Δ* cells. (A) chIP with anti-MYC from wildtype and *set1Δ* cells with either Sum1-MYC or Hst1-MYC expressed from their endogenous loci. A wild-type strain without an epitope tag (untagged) was used as a negative control. qPCR of immunoprecipitated DNA was performed with primers to amplify the promoter of *NDT80*, and percent input was determined as described in *Materials and Methods*. (B) chIP at additional loci for Sum1-MYC and (C) Hst1-MYC. The enrichment for either MYC-tagged protein is set at 1.0 for the wild-type strain, and relative enrichment for the *set1Δ* and untagged strains is shown. Percent input for chIP experiments is shown in Figure S4 in File S1. (D) Log_2_ fold-change of transcript levels for *SUM1*, *RFM1*, and *HST1* in *set1Δ* cells relative to wild type grown under vegetative conditions. These data were obtained from RNA-seq analysis (Jezek *et al.* 2017). (E) Immunoblotting of Sum1-MYC and Hst1-MYC in wild type and *set1Δ* cells using anti-MYC. anti-Hxk2 is shown as a loading control. (F) Venn diagram indicating shared differentially expressed genes in *set1Δ*, *sum1Δ*, *rfm1Δ*, and *hst1Δ* cells. *set1Δ* data were obtained from RNA-seq results (Jezek *et al.* 2017), and data for the other mutants was obtained from previously published microarray results of cells grown under similar conditions (Kemmeren *et al.* 2014). For all panels, error bars represent SEM of three biological replicates. Asterisks represent *p*-values from unpaired *t*-tests (* ≤ 0.05, *** ≤ 0.001; not significant is shown as ns).

We hypothesized that Set1 may specifically act to promote Sum1-Rfm1-Hst1 complex association with chromatin at middle sporulation loci. To test this, we performed chromatin immunoprecipitation (chIP) of MYC-tagged versions of Sum1 and Hst1 in wildtype and *set1Δ* cells under vegetative conditions. As a positive control, we analyzed association of Sum1-MYC and Hst1-MYC with the *NDT80* promoter, a well-described binding site for this complex (Figure 4A). We observed no significant difference in localization of either Sum1-MYC or Hst1-MYC to the promoter in *set1Δ* cells relative to wildtype, consistent with our finding that *NDT80* expression is similar to wildtype in *set1Δ* cells (Figure 2A). We next compared binding of Sum1-MYC and Hst1-MYC in wildtype and *set1Δ* cells to middle sporulation genes repressed by Set1, including *CDA1*, *LDS1*, and *SPR3* (Figure 4, B and C). At the promoter sequences, which contain the *MSE*s, there was a substantial decrease in both Sum1-MYC and Hst1-MYC binding in the absence of Set1. Although the decrease in Hst1-MYC in *set1Δ* cells at the *SPR3* promoter is not significant, it was reproducibly lower than wild type, and there was a significant decrease in Sum1-MYC binding at this region. At the ORF sequences queried, the 5′ end of *CDA1* was significantly depleted of Sum1-MYC and Hst1-MYC; however, no significant decrease was observed over the *SPR3* ORF. This difference is potentially linked to ncRNA-mediated regulation of *SPR3* (Margaritis *et al.* 2012), which is likely distinct from regulatory mechanisms of the other middle genes. Overall, these data support a role for Set1 in promoting the proper localization of Sum1 and Hst1 to middle sporulation genes.

One possible explanation for the decreased chromatin association of Sum1 and Hst1 in *set1Δ* cells is that the amounts of transcript or protein expressed are altered in the absence of Set1. We analyzed *SUM1*, *HST1*, and also *RFM1* mRNA levels in *set1Δ* cells, and no substantial change in expression under vegetative conditions was observed (Figure 4D). Immunoblotting of MYC-tagged Sum1 and Hst1 showed no difference in protein expression in *set1Δ* mutants (Figure 4E). These data indicate that Set1 does not regulate *SUM1*, *RFM1*, or *HST1* expression levels, and therefore the decreased chromatin localization of these proteins at middle genes is due to another regulatory function of Set1.

Interestingly, our chIP experiments suggest that Set1 is required for full binding of Sum1 and Hst1 at specific middle gene loci. For example, the Sum1-Rfm1-Hst1 complex also localizes to the *NDT80* promoter, although no binding changes at this gene were observed in *set1Δ* mutants (Figure 4A). We compared published transcriptomes of *set1Δ* cells and *sum1Δ*, *rfm1Δ* and *hst1Δ* mutants, and found that approximately half of genes regulated by this complex are also differentially expressed in *set1Δ* cells (Figure 4F). This suggests that Set1 promotes repression at a subset of middle sporulation genes targeted by Sum1-Rfm1-Hst1, but additional factors likely play a role in maintaining repression by this complex of other middle genes in the absence of Set1.

**Figure 5:**
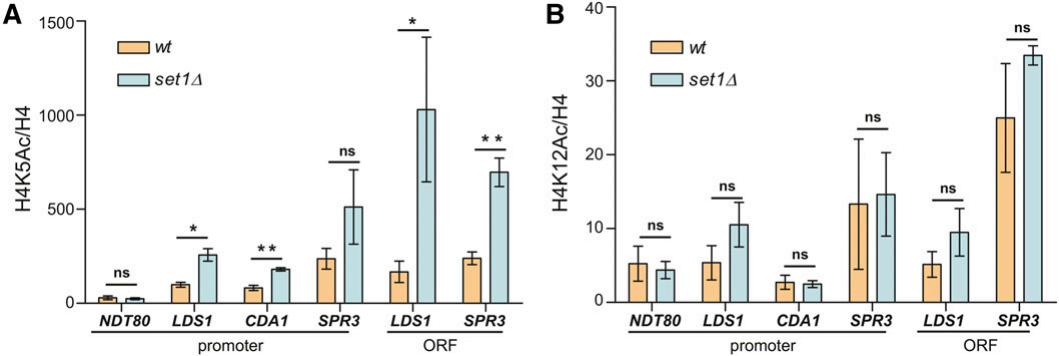
Cells lacking Set1 show increased H4K5 acetylation at middle sporulation loci. chIP was performed using antibodies against H4K5ac (A) and H4K12ac (B) in wildtype and *set1Δ* mutants. The indicated promoter and ORF sequences were probed by qPCR of the immunoprecipitated DNA. The percent input for the H4K5ac and H4K12ac chIPs was normalized to the percent input of total H4 at the same regions. Error bars represent SEM for three biological replicates. Asterisks represent *p*-values from unpaired *t*-tests (* ≤ 0.05, ** ≤ 0.01, not significant is shown as ns).

The specific decrease in Hst1 binding at middle sporulation genes suggested that these loci may have increased histone acetylation. To investigate this, the levels of H4K5ac and H4K12ac at these genes in wildtype and *set1Δ* cells were assayed using chIP. As expected, there was no change in H4K5ac at the *NDT80* promoter; however, we did observe increased levels of H4K5ac at some promoter and ORF sequences tested for the genes *CDA1*, *LDS1* and *SPR3* (Figure 5A). Interestingly, we did not see any substantial changes in H4K12ac levels at these loci in *set1Δ* mutants (Figure 5B). These observations are consistent with previous work indicating that Hst1 specifically targets H4K5ac, whereas it does not play much of a role in regulating H4K12ac levels or other H4 lysine acetylation sites (Weber *et al.* 2008). Together, these data suggest that Set1 promotes proper localization of Sum1 and Hst1 to middle sporulation genes under vegetative conditions, which is required to limit local acetylation at H4K5, likely promoting gene repression.

### Middle sporulation genes are marked by low levels of H3K4me2 and Set1 during vegetative growth

**Figure 6:**
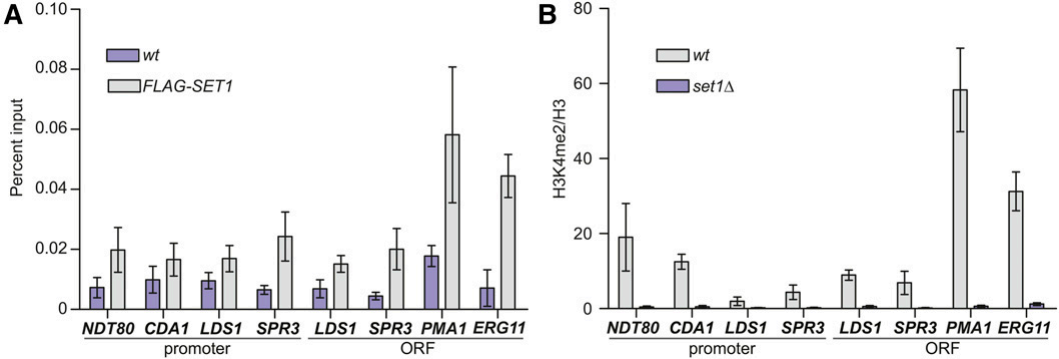
Set1 and H3K4me2 are not highly enriched at middle sporulation genes. (A) Anti-FLAG antibody was used for chIP from wildtype yeast or yeast expressing FLAG-Set1. qPCR of immunoprecipitated DNA was used to test enrichment of the indicated middle sporulation loci and positive control regions, the 5′ ORF of *PMA1* and *ERG11*. Percent input is shown for three biological replicates. Error bars represent SEM. (B) chIP of H3K4me2 in wildtype and *set1Δ* mutants at middle sporulation genes and positive control regions. The percent input for H3K4me2 was normalized to total H3 at the same regions. Error bars represent SEM for three biological replicates.

Set1 has been linked to gene repression through mechanisms that rely on its direct action at specific genes, such as ergosterol biosynthetic genes (South *et al.* 2013), or indirectly by its actions in other genomic regions, as proposed at telomeres (Santos-Rosa *et al.* 2004; Venkatasubrahmanyam *et al.* 2007). We therefore tested whether or not Set1 is directly associated with middle sporulation genes using chIP of N-terminally FLAG-tagged Set1. qPCR of the 5′ end of the ORF for two genes known to be direct targets of Set1 (*PMA1* and *ERG11*) showed clear enrichment of FLAG-Set1; however, the chromatin regions we identified as depleted for Sum1-MYC and Hst1-MYC showed low-to-moderate levels of FLAG-Set1 (Figure 6A). We also monitored H3K4me2 levels at these loci and observed lower levels of this mark at middle sporulation genes than *PMA1* and *ERG11*. Given the relatively low level of Set1 and the methyl mark, these data suggest that the maintenance of Sum1 and Hst1 at specific middle sporulation genes may be due to functions of Set1 associated with other genomic regions, or, alternatively, that low levels of Set1 and H3K4me2 at middle genes may be sufficient to maintain repression, as discussed further below.

## Discussion

This work describes an unexplored role for Set1 and methylation at H3K4 in repressing genes required for the meiotic program in yeast during growth under nutrient-rich conditions. Our findings support a model in which Set1 promotes association of the locus-specific Sum1-Rfm1-Hst1 repressor complex with middle sporulation genes to maintain deacetylation primarily at H4K5 during vegetative growth. Broadly, our results indicate that Set1 and H3K4 methylation promote a repressive chromatin state at specific loci by modulating the binding of a specialized transcription factor complex. Furthermore, it suggests that Sum1 complex binding to middle sporulation genes is dependent not just on DNA sequence elements, but also on histone modification status.

### New roles for Set1 and H3K4 methylation in chromatin regulation of middle sporulation genes

Repression of middle sporulation genes under vegetative conditions is well-characterized to be dependent on a sequence element within their promoters, the *MSE*, which is recognized and bound by Sum1 (Ozsarac *et al.* 1997; Xie *et al.* 1999). However, our data suggest that histone methylation at H3K4 also promotes Sum1 binding to middle sporulation loci, indicating that there are additional chromatin elements that contribute to properly localizing this transcription factor. Although a previous screen of histone residues required for Sum1-mediated repression did not uncover a specific role for H3K4, this residue was not tested in isolation and specific reporter genes were used, which may not reflect all middle sporulation genes (Prescott *et al.* 2011). Interestingly, however, Set1 was required for gene silencing mediated by a gain-of-function mutant in *SUM1*, known as *SUM1-1*, although this appeared to be independent of Sum1-1 recruitment to silent regions (Prescott *et al.* 2011).

Notably, we did not observe Set1-mediated repression at all genes regulated by the Sum1 complex. This was evident in genome-wide comparisons of differentially expressed genes in cells lacking either Set1 or Sum1, Rfm1, or Hst1 (Figure 4F) and in our targeted gene expression and chIP analysis. In particular, both *NDT80* and the early-middle gene *SMK1* are repressed by Sum1-Rfm1-Hst1 under vegetative conditions; however, they are not derepressed in *set1Δ* cells (Figure 2), nor is there a loss of Sum1 or Hst1 binding at the *NDT80* promoter (Figure 4A). This suggests that Set1 does not broadly disrupt Sum1 or Hst1 chromatin association, but rather that its role in repression results in gene-specific changes. In addition to previous reports suggesting that Sum1 may have context-dependent roles at different promoters (Xie *et al.* 1999; McCord *et al.* 2003; Pierce *et al.* 2003), our findings are further indication that there are multiple, overlapping mechanisms that repress middle sporulation genes in the absence of meiotic signals, and that expression of these genes is likely to be differentially regulated by the cell, despite sharing common promoter elements and temporal expression patterns.

### Set1 promotes gene repression in a locus-specific manner

While Set1 and H3K4 methylation are cotranscriptionally recruited and deposited, respectively, and linked to active transcription (Bernstein *et al.* 2002; Santos-Rosa *et al.* 2002; Ng *et al.* 2003; Liu *et al.* 2005; Pokholok *et al.* 2005), a number of studies have identified potential mechanisms by which Set1 and H3K4 methylation promote transcriptional repression. For example, Set1-dependent silencing of subtelomeric genes has been proposed to be a consequence of Sir protein redistribution to euchromatic regions (Santos-Rosa *et al.* 2004; Venkatasubrahmanyam *et al.* 2007), or due to links between Set1 and telomere maintenance pathways (Corda *et al.* 1999; Margaritis *et al.* 2012; Jezek *et al.* 2017). At other loci, H3K4me2 and H3K4me3 are associated with HDAC recruitment and subsequent gene repression, such as with the HDAC-containing Set3C (Kim and Buratowski 2009; Kim *et al.* 2012), and Set1-dependent Rdp3L localization to ribosomal biogenesis genes (Weiner *et al.* 2012). Furthermore, Set1 has been shown to promote noncoding transcription, particularly antisense stable or cryptic noncoding RNAs (ncRNAs), at multiple classes of genes. Depending on the locus, it is either the noncoding transcript, or the act of transcription itself, that represses the coding transcript, such as through the deposition of the H3K4me2 mark and the subsequent recruitment of the Set3 complex (Camblong *et al.* 2009; Pinskaya *et al.* 2009; Kim *et al.* 2012; Margaritis *et al.* 2012; Castelnuovo *et al.* 2014).

**Figure 7:**
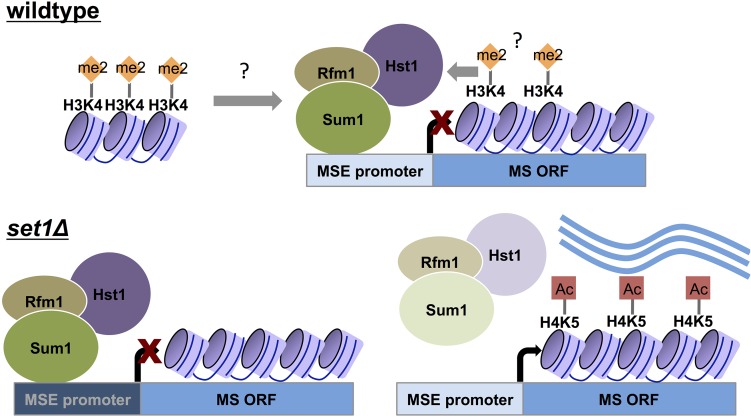
Model of Set1-dependent repression of middle sporulation genes. In wild-type cells grown under vegetative conditions, Set1, and other machinery required for H3K4 methylation, promote full binding of the Sum1-Rfm1-Hst1 complex to middle sporulation genes, repressing their expression. Based on the levels of Set1 and H3K4me2 at middle genes, we propose two possible models for how Set1 generates a chromatin landscape that restricts Sum1 binding to *MSE*s. The low levels of Set1 and H3K4me2 at middle genes may be sufficient to directly promote Sum1-Rfm1-Hst1 association at these loci. Alternatively, dimethylation of H3K4 at other genomic regions may indirectly prevent spurious Sum1-Rfm1-Hst1 binding throughout the genome, thereby restricting it to middle sporulation genes. In *set1Δ* cells, some *MSE*-regulated genes maintain Sum1-Rfm1-Hst1 (dark blue promoter, *e.g.*, *NDT80*), whereas others lose the complex (light blue promoter). We postulate that middle sporulation genes with *MSE*s that have lower affinity for Sum1 also require Set1 and H3K4 methylation to retain the complex. Those genes with decreased levels of Sum1-Rfm1-Hst1 complexes are also characterized by increased acetylation at H4K5 and increased gene expression.

Our findings suggest a model in which Set1 and the H3K4me2 mark promote binding of the Sum1-Rfm1-Hst1 complex to a subset of middle sporulation genes, causing derepression in the absence of Set1-catalyzed methylation (Figure 7). A specific increase in H4K5ac is observed at these regions, likely due to the loss of local Hst1, which has been shown to preferentially target H4K5 for deacetylation at chromatin (Weber *et al.* 2008). ChIP experiments indicate that H3K4me2 and Set1 have relatively low abundance at middle gene loci compared to previously identified direct targets of Set1. We postulate that either H3K4me2 at other genomic locations helps to restrict Sum1 binding to a subset of middle sporulation genes, or relatively low levels of Set1 and H3K4me2 at middle genes are sufficient to directly promote repression (Figure 7). This would be potentially analogous to the low levels of H3K36 methylation implicated in repression of weakly transcribed genes by Set2 (Li *et al.* 2007). Furthermore, it may be that Set1 activity is most important for middle genes that have relatively weak *MSE*s for stabilizing Sum1-Rfm1-Hst1 association (Xie *et al.* 1999; Pierce *et al.* 2003). Direct comparison of *MSE*s using expression reporter assays and electrophoretic mobility shift assays with Sum1 protein indicated that one of the two *MSE*s from the *NDT80* promoter induces greater repression and is a stronger binding partner for Sum1 than an *MSE* from the *SPR3* promoter (Xie *et al.* 1999), which showed reduced Sum1 binding in the absence of Set1 in our chIP assays. Further investigation is warranted to determine if the chromatin landscape in *set1Δ* is unable to restrict binding of Sum1-Rfm1-Hst1 to weaker *MSE*s, resulting in its partial disassociation from chromatin and subsequent derepression of this subset of middle sporulation genes.

Another possible component of Set1-dependent silencing of middle sporulation genes is noncoding transcription at these loci. We inspected published tiling microarray data of middle sporulation genes from cells lacking the nuclear exosome components Rrp6 and Dis3 (Gudipati *et al.* 2012), which function in RNA degradation and promote stabilization of cryptic unstable transcripts and stable unannotated transcripts (Davis and Ares 2006; Schneider *et al.* 2012). Of the most derepressed middle sporulation genes identified by RNA-seq in *set1Δ* cells (log_2_ fold-change ≥2), only two out of 17 genes showed ncRNA transcripts, both encoded in the promoters. Of the genes analyzed by qPCR in this study, *CDA1*, *LDS1* and *SGA1* do not show antisense or adjacent sense ncRNAs, whereas *SPR3*, *LOH1*, *SMA1* and *NDT80* do have ncRNA transcripts. While it still may be possible that part of the mechanism of Set1-mediated repression of middle sporulation genes relies on noncoding transcription, these observations did not indicate a clear correlation between Set1-dependent repression and ncRNA production. Additionally, we eliminated the potential role of the Set3 HDAC complex, which is associated with cryptic transcription at Set1-regulated genes (Kim and Buratowski 2009; Kim *et al.* 2012), in middle sporulation gene repression (Figure S2 in File S1). While it remains possible that Set1-dependent repression at these loci is associated with cryptic or antisense transcription, such as previously demonstrated at *SPR3* (Margaritis *et al.* 2012), it is unlikely to be a unifying principle linking the repression of these genes to Set1 and H3K4 methylation.

The repression of meiotic-specific transcripts during mitotic growth serves to prevent aberrant gene expression patterns that may promote genomic instability (Janic *et al.* 2010; Folco *et al.* 2017). Interestingly, the role for Set1 in middle sporulation gene repression investigated here appears largely distinct from its functions during meiosis, as Set1 does not repress these genes in nonfermentable carbon sources or during the early stages of the sporulation program (Figure 3). Further investigation will be required to link the functions of Set1 and H3K4 methylation in mitotically dividing yeast *vs.* meiotic cells, or under nonfermentable *vs.* respiratory conditions. Nonetheless, our findings identify an unexplored role for Set1 and H3K4 methylation in the repression of meiotic differentiation genes in yeast, and provide further evidence that global chromatin modifiers can promote localized control of gene expression through locus-specific regulatory proteins.

## Supplementary Material

Supplemental material is available online at www.g3journal.org/lookup/suppl/doi:10.1534/g3.117.300150/-/DC1.
