# Supplementary material for: Repression of Middle Sporulation Genes in Saccharomyces cerevisiae by the Sum1-Rfm1-Hst1 Complex Is Maintained by Set1 and H3K4 Methylation
Source: G3 (Bethesda). 2017 Oct 24;7(12):3971–82. doi: 10.1534/g3.117.300150 (PMC5714494; doi:10.1534/g3.117.300150)
Supplement: Supplementary file 1 [file 3971FileS1.pdf]

SUPPLEMENTAL MATERIALS

Jaiswal *et al.*

Repression of middle sporulation genes in *Saccharomyces cerevisiae* by the Sum1-Rfm1-Hst1 complex is maintained by Set1 and H3K4 methylation

Figure S1
Figure S2
Figure S3
Figure S4
Table S1
Supplemental References

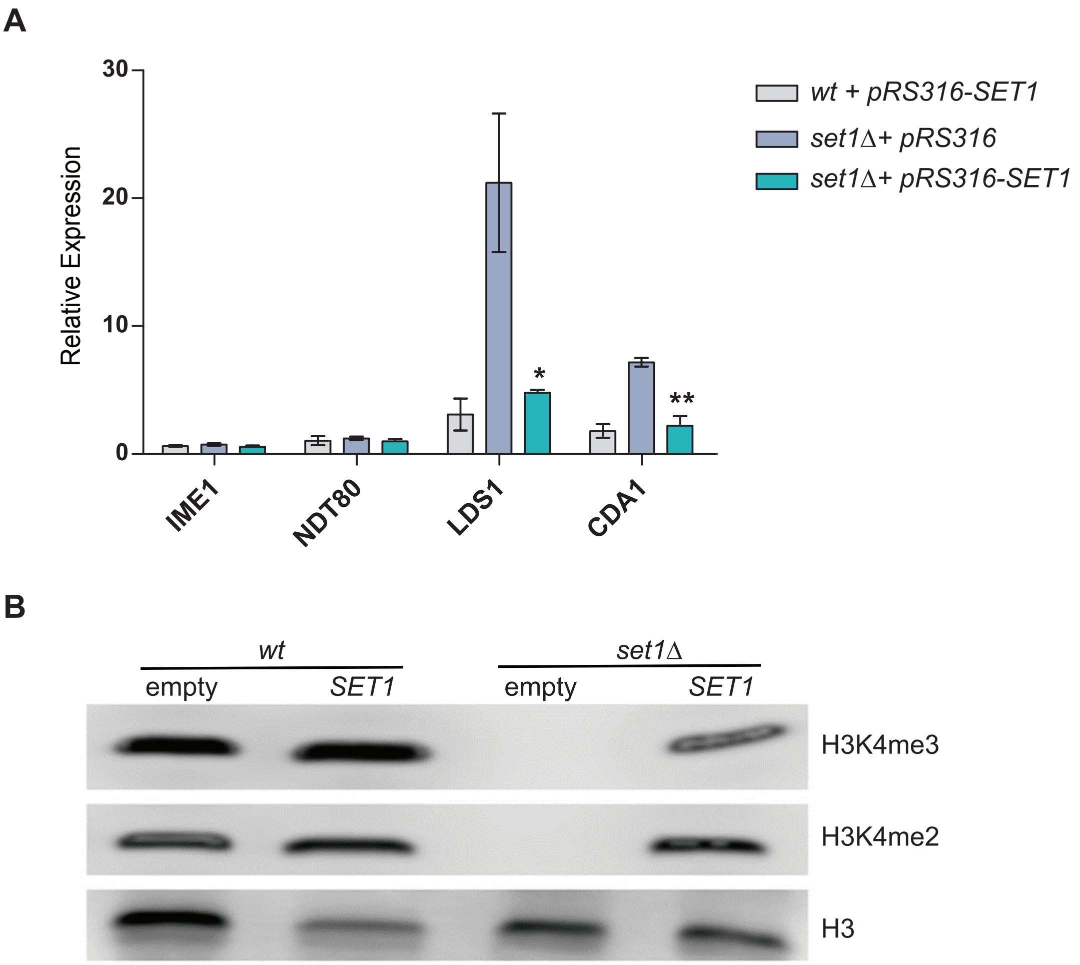

Figure S1. Expression of episomal *SET1* in *set1Δ* cells rescues the defect in middle sporulation gene repression. (**A**) Expression of early and middle genes by qRT-PCR in wildtype and *set1Δ* cells carrying either *pRS316-SET1* or an empty *pRS316* vector. Cells were grown in synthetic complete medium with glucose and lacking uracil. *pRS316-SET1* expresses *SET1* under the control of its endogenous promoter. Expression levels were normalized to the control gene *TFC1*. Error bars represent SEM for three biological replicates. Asterisks represent *p*-value (* < 0.05; ** < 0.01; *** < 0.001) from unpaired t tests comparing *set1Δ* with *pRS316-SET1* to *set1Δ* with *pRS316*. (**B**) Western blotting of whole cell extracts from wildtype and *set1Δ* cells carrying either *pRS316-SET1* or an empty *pRS316*. Antibodies recognizing H3K4me3, H3K4me2 and H3, as loading control, were used.

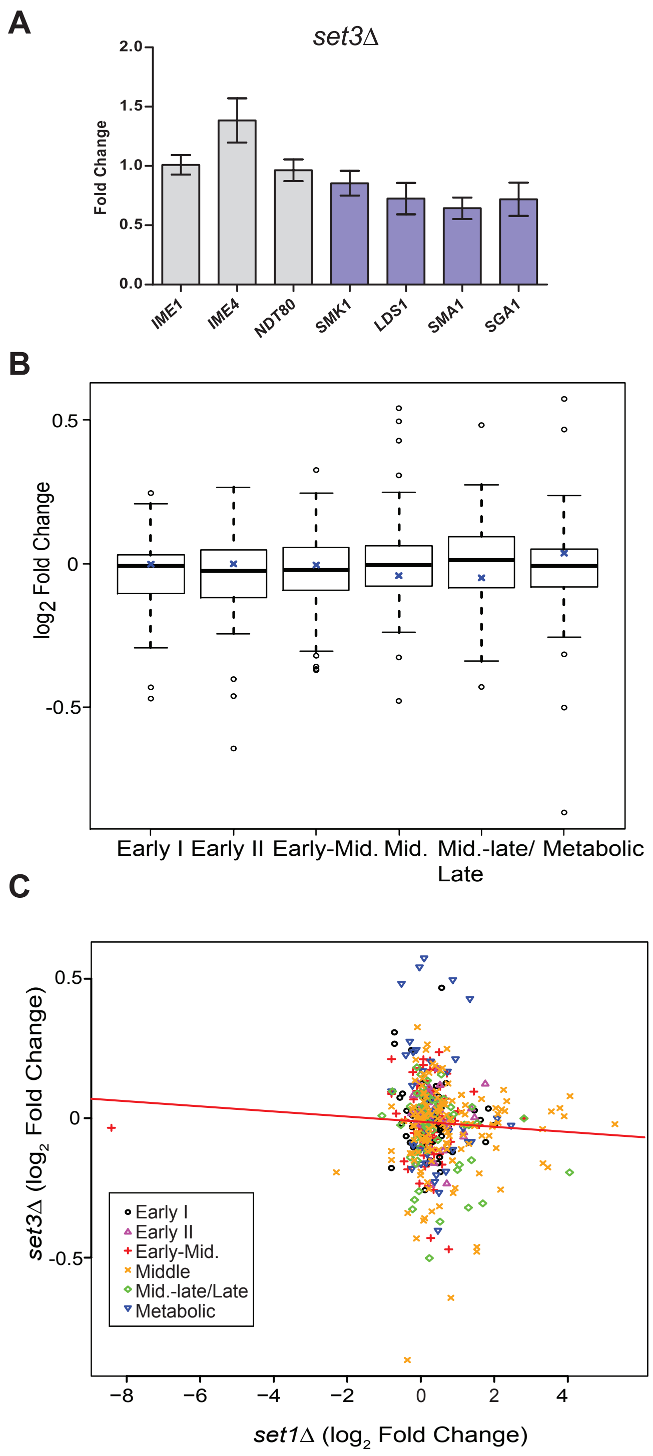

Figure S2. Set1-mediated repression of middle sporulation genes does not depend on Set3. (**A**) qRT-PCR of mRNA levels of early (gray) and middle (purple) sporulation genes in *set3Δ* cells. Data are presented as described for Figure 2. (**B**) Log_2 fold-change of genes from *set3Δ* cells belonging to each of the indicated sporulation classes, as previously defined (CHU *et al.* 1998). Genome-wide expression values for

set3Δ cells were obtained from previously-published microarray results (KEMMEREN *et al.* 2014). Data are presented as described for Figure 1C. (**C**) Linear regression model of $\log_2$ fold-change expression values for *set1Δ* relative to *set3Δ* mutants for genes that encompass each of the sporulation classes.

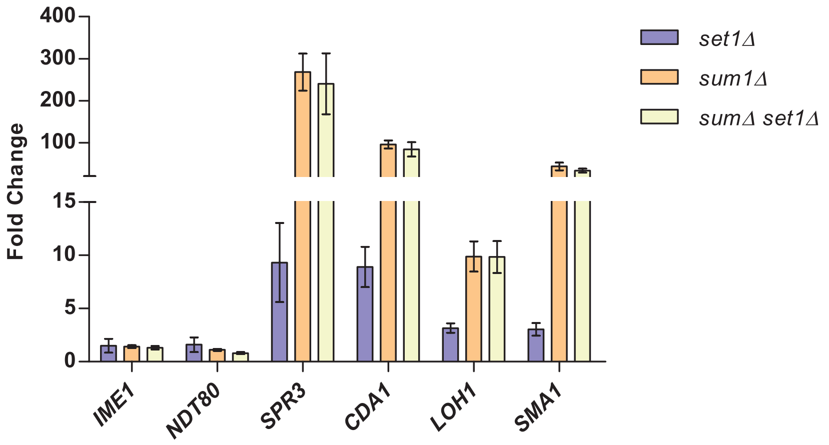

Figure S3. Middle sporulation gene expression in *set1Δ* and *sum1Δ* single and double mutants. Expression of early and middle genes by qRT-PCR in wildtype and *set1Δ*, *sum1Δ* and *set1Δ sum1Δ* cells. Expression levels were normalized to the control gene *TFC1*. Error bars represent SEM for three biological replicates.

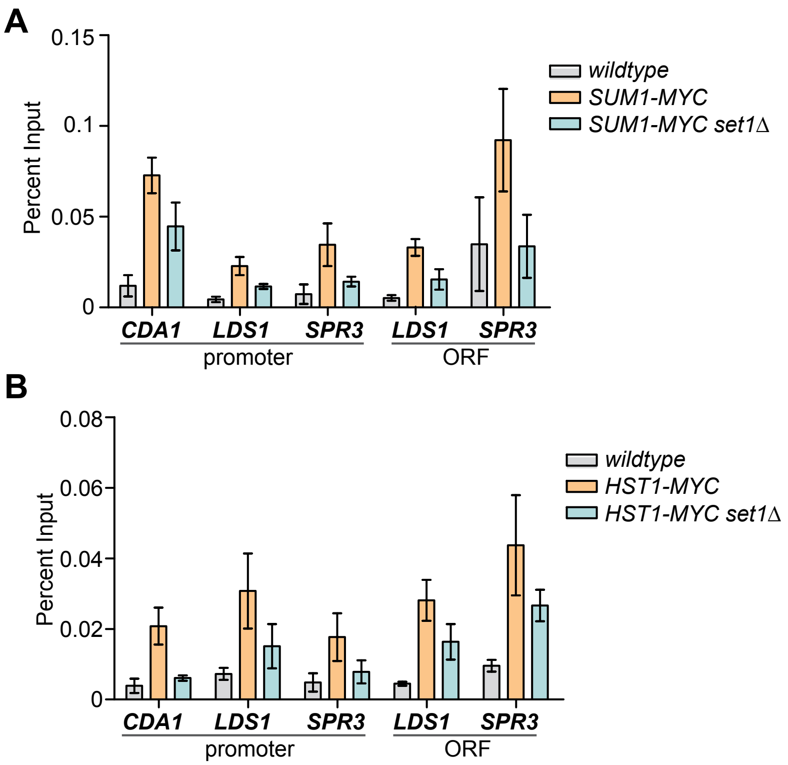

Figure S4. chIP of Sum1-MYC and Hst1-MYC in wildtype and *set1Δ* cells at middle sporulation genes. chIP with anti-MYC from wildtype and *set1Δ* cells with either Sum1-MYC or Hst1-MYC expressed from their endogenous loci. A wildtype strain without an epitope tag was used as a negative control. qPCR of immunoprecipitated DNA was performed with primers to amplify the promoter or ORF sequences as indicated. Percent input was determined as described in Materials and Methods. Error bars represent SEM of three biological replicates.

Table S1. Primers used in this study.

Oligo #	Experiment	ORF/Position	Sequence
oEG907	GEX	IME1 F	GCAACTGGTCCTGAAAGAGG
oEG908	GEX	IME1 R	GTGGAACGTAGATGCGGATT
oEG905	GEX	IME4 F	AAAACTGGAACCAGCCACAC
oEG906	GEX	IME4 R	TCCAATACTGCTGCTGATGC
oEG897	GEX	NDT80 F	ACCTTTCATGGGGAGAATCC
oEG898	GEX	NDT80 R	GAGGCGTTTTCATTTCTTGC
oEG899	GEX	SMK1 F	TGTCATCCACCGAGATTTGA
oEG900	GEX	SMK1 R	ACCCCTAGCGAGACCAAAAT
oEG901	GEX	LDS1 F	TTTGCCCTGGTAAGCTTTGT
oEG902	GEX	LDS1 R	GGCAATGAAAAGCCCAATAG
oEG959	GEX	CDA1 F	GGCAAACATTTCCCCAAGTA
oEG960	GEX	CDA1 R	CCCACAAGACAACCGTTAGG
oEG963	GEX	SPR3 F	TCTGGATTCGCTGAGGAAGT
oEG964	GEX	SPR3 R	TTTCAGTTCAGGGCTTTTCG
oEG961	GEX	LOH1 F	CTCAAGGAGTGGCCGATTTA
oEG962	GEX	LOH1 R	TATGAAACCGATGCTTCCAA
oEG903	GEX	SMA1 F	AGTTCCATTTAGGCCCTGGT
oEG904	GEX	SMA1 R	TGCCGCGTTAGTAAAAGACC
oEG797	GEX	SGA1 F	GCTTCCCATCTTCCGTTCGA
oEG798	GEX	SGA1 R	ACCCGTTGCATTTTTGGAGC
oEG543	GEX	TFC1 F	ACACTCCAGGCGGTATTGAC
oEG544	GEX	TFC1 R	CTTCTGCAATGTTTGGCTCA
oEG936	ChIP	LDS1 promoter F	TGGAAAAGGAAGTTTCAAATCA
oEG937	ChIP	LDS1 promoter R	CCTGTTGTTGTTATTGTTTGTATAATG
oEG938	ChIP	LDS1 5' ORF F	GGAGGCCAATCGTATGAAAA
oEG939	ChIP	LDS1 5' ORF R	CACGTATCTGGTACGGCTCA
oEG965	ChIP	CDA1 promoter F	TTTTCCAGATGTCACATTTTCC
oEG966	ChIP	CDA1 promoter R	CAACCCACTTGTTGGTATTTTTC
oEG967	ChIP	SPR3 promoter F	TGGTAGTGGAAAGAAAAGGATCA
oEG968	ChIP	SPR3 promoter R	TCAATGACGCAAAAGGAATTT
oEG893	ChIP	NDT80 promoter F	CTGACAAAGCTCCAGAACGGT
oEG894	ChIP	NDT80 promoter R	AGGGACCTTGGCTTTTCGAA
oEG153	ChIP	PMA1 5' ORF F	TCAGCTCATCAGCCAACTCAAG
oEG154	ChIP	PMA1 5' ORF R	CGTCGACACCGTGATTAGATTG
oEG568	ChIP	ERG11 5’ ORF F	CCTCTTATTCCGTCGGTGAA
oEG569	ChIP	ERG11 5’ORF R	TGTGTCTACCACCACCGAAA

The experiment type, either for qPCR analysis of gene expression (GEX) or chromatin immunoprecipitation (ChIP) is listed for each primer. Promoter or ORF location is indication for chIP primers. Sense primers are designated forward (F) and antisense primers as reverse (R).

Supplemental References

Chu, S., J. DeRisi, M. Eisen, J. Mulholland, D. Botstein *et al.*, 1998 The transcriptional program of sporulation in budding yeast. Science 282**:** 699-705.

Kemmeren, P., K. Sameith, L. A. van de Pasch, J. J. Benschop, T. L. Lenstra *et al.*, 2014 Large-scale genetic perturbations reveal regulatory networks and an abundance of gene-specific repressors. Cell 157**:** 740-752.
